# Supplementary material for: The Effect of Starting Blood Glucose Levels on Serum Electrolyte Concentrations during and after Exercise in Type 1 Diabetes
Source: Int J Environ Res Public Health. 2023 Jan 24;20(3):2109. doi: 10.3390/ijerph20032109 (PMC9915529; doi:10.3390/ijerph20032109)
Supplement: Supplementary file 1 [file ijerph-20-02109-s001.zip › ijerph-2182241-supplementary.pdf]

Supplementary Table S1. Serum and urine measures in CON, T1D MOD, and T1D HI presented as mean ± SEM.

		**CON (n=12)**	**T1D MOD (n=12)**	**T1D HI (n=12)**
Serum Glucose (mmol/L)	Pre exercise (0 Time)	4.9 ± 0.1	10.4 ± 0.4	13.7 ± 0.7
	Post exercise (45 min)	5.08 ± 0.2 *# ^	7.5 ± 0.7	10.1 ± 0.6 *#
	Recovery (105 min)	4.7 ± 0.05 *# ^	11.2 ± 0.8	14.0 ± 1.3 *#
Serum Insulin (mmol/L)	Pre exercise (0 Time)	54.0 ± 14.0	169.6 ± 50.0	167.4 ± 45.3
	Post exercise (45 min)	21.4 ± 4.7 *^	183.7 ± 61.3	164.6 ± 49.8
	Recovery (105 min)	26.1 ± 5.2 *	168.2 ± 54.2	167.9 ± 41.0
Serum Sodium (mmol/L)	Pre exercise (0 Time)	141.3 ± 0.2	140.6 ± 0.5	139.8 ± 0.6
	Post exercise (45 min)	141.7 ± 0.2 # ^	143.0 ± 0.7	141.8 ± 0.5 #
	Recovery (105 min)	141.2 ± 0.4 # ^	139.6 ± 0.7	139.1 ± 0.4 #
Serum Potassium (mmol/L)	Pre exercise (0 Time)	3.7 ± 0.1	3.9 ± 0.09	4 ± 0.08
	Post exercise (45 min)	4.3 ± 0.04 #	4.3 ± 0.08	4.5 ± 0.07 #
	Recovery (105 min)	4 ± 0.1 #	4 ± 0.08	4.1 ± 0.1 #
Serum Calcium (mmol/L)	Pre exercise (0 Time)	2.1 ± 0.1	2.2 ± 0.02	2.2 ± 0.03
	Post exercise (45 min)	2.4 ± 0.01 #	2.3 ± 0.04	2.4 ± 0.03 #
	Recovery (105 min)	2.2 ± 0.01 *#	2.2 ± 0.02	2.2 ± 0.02 #
Serum Magnesium (mmol/L)	Pre exercise (0 Time)	0.8 ± 0.01	0.7 ± 0.01	0.7 ± 0.01
	Post exercise (45 min)	0.8 ± 0.01 *#	0.7 ± 0.01	0.7 ± 0.01
	Recovery (105 min)	0.7 ± 0.01 *#	0.6 ± 0.02	0.7 ± 0.01 #
Urine Glucose Concentration (mmol/L)	Pre exercise (0 Time)	0.07 ± 0.01	2.9 ± 1.1	4.2 ± 1.2
	Post exercise (45 min)	0.08 ± 0.01 *	1.7 ± 0.8	7.3 ± 0.7 *^
	Recovery (105 min)	0.1 ± 0.02 *	1.9 ± 0.9	5.9 ± 0.9 *
Urine Specific Gravity	Pre exercise (0 Time)	1.013 ± 0.002	0.018 ± 0.002	1.018 ± 0.003
	Post exercise (45 min)	1.012 ± 0.002 *	1.021 ± 0.002	1.020 ± 0.002
	Recovery (105 min)	0.014 ± 0.002 *	1.021 ± 0.002	1.020 ± 0.002
Urine Sodium Concentration (mmol/L)	Pre exercise (0 Time)	85.2 ± 14.6	75.3 ± 14.7	61.3 ± 12.4
	Post exercise (45 min)	58.1 ± 6.4	79.0 ± 13.5	58.6 ± 8.4
	Recovery (105 min)	81.4 ± 14.1 #	85.2 ± 11.2	74.3 ± 7.8
Urine Potassium Concentration (mmol/L)	Pre exercise (0 Time)	46.6 ± 8.5	77 ± 12.3	56.5 ± 7.6
	Post exercise (45 min)	43.1 ± 6.1 *	86.9 ± 9.9	62.7 ± 8.1
	Recovery (105 min)	40.6 ± 5.6 *	78.8 ± 13.9	57.6 ± 9.3
Urine Calcium Concentration (mmol/L)	Pre exercise (0 Time)	3.3 ± 0.8	2.8 ± 0.6	2.3 ± 0.6
	Post exercise (45 min)	2.3 ± 0.5	2.9 ± 0.6	1.9 ± 0.3
	Recovery (105 min)	2.8 ± 0.7	2.5 ± 0.4	1.8 ± 0.3

* Significant effect of treatment, # significant effect of time, and ^ significant interaction from pre exercise to post exercise and post exercise to recovery for CON vs. MOD and MOD vs. HI compared by ANOVA.
